# Health-promoting prisons in the female estate: an analysis of prison inspection data

**DOI:** 10.1186/s12889-021-11621-y

**Published:** 2021-08-21

**Authors:** James Woodall, Charlotte Freeman, Louise Warwick-Booth

**Affiliations:** School of Health and Community Studies, Leeds Metropolitan University, Leeds, England

## Abstract

**Background:**

Women in prison have comparatively greater health needs than men, often compounded by structures and policies within the prison system. The notion of a ‘health-promoting’ prison is a concept which has been put forward to address health inequalities and health deterioration in prisons. It has, however, not been fully discussed in relation to women in prison. The paper aims to distil the learning and evidence in relation to health promotion in female prisons using prison inspection reports of women’s prisons in England and Wales.

**Methods:**

Prison inspection reports are one way of ascertaining the contemporary situation in prisons. Prison inspections are often unannounced and use a myriad of methods to draw conclusions around various aspects of prison life. Thirteen prison inspection reports were analysed thematically focusing on health promotion within the institutions. Two analysts conducted the work using NVivo 12.

**Results:**

Five core thematic areas were identified during the analysis of the reports. Saliently, a joined-up approach to health promotion was not a common feature in the prisons and indeed the focus tended to be on screening and ‘lifestyle issues’ rather than a concern for the underlying determinants of health. There was often an absence of a strategic approach to health promotion. There were some good examples of the democratic inclusion of women in prison in shaping services, but this was not widespread and often tokenistic. There were some examples of inequity and the inspection reports from a small number of institutions, illustrated that the health needs of some women remained unmet.

**Conclusions:**

The paper suggests that there is potentially some work before conditions in women’s prisons could be described as ‘health-promoting’, although there are some examples of individual prisons demonstrating good practice. The health promoting prison movement has, implicitly at least, focused on the needs of men in prison and this has been to the exclusion of the female prison population. This does lead to several challenges and the potential for exacerbating health challenges faced by an already marginalised and vulnerable group. Greater focus on the health promotion needs of women in prison is required.

## Introduction

There is much endorsement for policy and practice that reconfigures prisons as a ‘health-promoting’ rather than ‘health depleting’ environment. Much of this endorsement has arisen in Europe and has been taken forward to greater or lesser extent in various countries. In some countries there has been little momentum for this [1–5], but in England and Wales [6], Scotland [7] and some other countries in Western Europe there has been some commitment to developing the theory and practice. The concept of a health promoting prison is not new – conceptually it is a sound theoretical idea that suggests how prisons should be salutogenic, supportive and empowering and work toward addressing the wider determinants of health. This includes all constituents of the prison, including people in prison; their families; staff; and the wider community. In practice, however, the health promoting prison has been criticised and challenged with many outlining the difficulty of aligning health promotion values in a prison context and concluding that it is somewhat of an oxymoron [8]. Some of the critique is indeed valid, given that the health promoting prison has lacked any rigorous evaluation [9]. Moreover while health promotion has been an integral part of the prison regime, it has generally focussed on disease prevention and screening rather than on enhancing health or improving well-being [6]. This is perhaps understandable given the limited resource and the importance of maintaining a safe environment, free from disease. Indeed, epidemiological data consistently shows how prevalent certain diseases are in prison in comparison to the general community.

The focus on prisons as a setting for health has derived more broadly from the ‘settings approach’ in health promotion. The key idea of the settings approach, or healthy settings approach is that investments in health are made in social systems where health is not their primary remit [10] and areas such as schools and workplaces have had a long-standing track-record with this approach. The health promoting prison has, at least implicitly, been focussed on the needs of men. This may be understandable, given that men represent the overwhelming majority of people in prison. For example, across the UK less than 5% of people in prison are women [11] . Despite this, women are in prison and, compared to men, have greater health needs – as an example, more than two-thirds of women (67%) reported that they had a mental health problem compared with over two-fifths of men (43%) [12]. In addition, women are significantly more likely to say they have a problem with alcohol on arrival at prison than men [12] and tend to be particularly marginalised and excluded with histories of victimization or abuse, substance use, mental health problems, and traumatic relationships [13]. Women in prison tend to also be located at a far greater distance from their home than men in prison and tend to be the primary caregiver – this can lead to reduced visitation from families because of excessive and costly journeys [14]. Issues can be yet further compounded: women’s prisons can resemble men’s prisons in terms of prison management styles, staffing techniques and the programming of services [13]. Moreover, a recent review found that gender-sensitive policies across the criminal justice system were failing women, with ‘male-dominated’ spaces contributing to further challenges in relation to rehabilitation and health [15].

There is a strong rationale for more gender-sensitive policy and practice in relation to the way prisons are run and managed [15], but perhaps none more so than when considering aspirations to create health-promoting prisons. Given health promotion has core values relating to equity [9] it seems an oversight not to have considered women in prison in greater detail. Several commentators have also argued that there remains considerable gender insensitivity in policies, procedures and practices [16]. Healthcare needs of incarcerated pregnant women, as a specific example, are often left unmet, with negative experiences of antenatal care, intensified by the loss of control over their pregnancies [17]. When a state deprives individuals of their liberty, it takes on the mandate to oversee their welfare. People in prison have no choice but to rely on the state to protect and promote their health [18]. In recent years, several Member States in the WHO European Region have transferred the responsibility for prison health to their health ministry, though integrating prison health into national health systems has proven to be a long and sometimes challenging process. For example, in England and Wales the process was started in 2000 and was not completed until 2006 [19].

Basic standards of care for women in prison is broadly lacking and, it seems that, gender-specific prison facilities have become an aspiration rather than a reality [4]. Baroness Corston in 2007 focused attention on ‘women with particular vulnerabilities in the criminal justice system’ and highlighted the needs of women prisoners. The report made a series of recommendations about how women’s prisons could be improved [20]. This gathered momentum, but health outcomes for women in prison have since remained poor more than a decade on [21].

Prison inspection reports are one way of ascertaining the contemporary situation in prisons [6, 7, 22]. Independent prison inspections, conducted by Her Majesty’s Inspectorate of Prisons for England and Wales (HMIP), form one element in how prisons are assessed in relation to evaluating the provision of health and well-being initiatives [23]. The view provided by HMIP is highly-valuable, given that they have access to all areas of an institution and can arrive unannounced [24]. Prison inspections draw on a range of data, including a confidential survey of a representative proportion of the prisoner population; prisoner focus groups; individual interviews carried out with staff and prisoners; documentation analysis; and observation by inspectors [23]. It is a methodology which is recognised for its international excellence [25] and having ‘influence [that] is so pervasive that the HMIP can be said indirectly to regulate prison conditions’ in England and Wales ([26] , p.532]). This paper is timely given the increased scrutiny of regulation and inspection in prisons, particularly in relation to health and well-being. A recent report in 2018 by the House of Commons Health and Social Care Committee [27] outlined a lack of definitional clarity used by HMIP in relation to a ‘a whole prison approach’ and suggested that recommendations from inspections were infrequently acted upon by the Prison Service. The report signalled a renewed emphasis that HMIP must be listened to and acted on to improve prisoner health outcomes [27], thus reasserting the pivotal role of HMIP in shaping policy and practice. The paper aims to distil the learning and evidence in relation to health promotion derived from the inspections of women’s prisons in England and Wales. This is a tested methodology [6, 7, 22] to assessing the progress of prisons using publicly-available, independent examination of the female prison estate.

## Methodology

Documentary analysis is a well-established way of gathering data and can take a myriad of forms. The approach can include the systematic analysis of organisational and institutional reports [28]. In understanding how prisons can be configured to be more health-enhancing than depleting, prison researchers have largely neglected documentary methods, opting instead to pursue empirical approaches [29–31]. These studies have offered a great deal, but have tended to focus on a single prison or small numbers of prisons which makes transferability of findings challenging given the heterogeneity of most prisons. Using a more cross-sectional approach provides wider insight into activities designed to ‘promote health and well-being’ in prison establishments in England and Wales identified by HMIP.

Prison inspectors work to a set of “expectations” relating to the level and quality of service that it expects to find in prisons [20, 32]. It is, in effect, the criteria by which prisons are assessed and evaluated. The public repository hosting the HMIPS reports was accessed via the internet. As this repository contains the full range of inspection reports carried out by HMIPS since 2012 the relevant reports were identified by filtering by the name of each institution within the female estate. The most recent unannounced inspection report for each institution was downloaded. Because of the limited number of inspection reports available for analysis, no further sampling was undertaken and all of the existing thirteen reports were included in the analysis. The inspection reports were downloaded during December 2020 and included reports published between 2017 and 2020. To target the Inspectorate’s reporting of the promotion of health and wellbeing, a subsection of the report was focused on. Section two of the report describes the second healthy prison test of ‘Respect’. This contains the subsection ‘Health services’ and within this section, under the ‘Governance arrangements’ heading, HMIP state the expectation that the promotion of physical and mental health will be demonstrated by the prison [20]. Consequently, this section was the unit of analysis for each of the thirteen reports.

The standards of treatment and conditions that the prisons were expected to achieve included:

- Women are cared for by a health service that accurately assesses and meets their health needs while in prison and which promotes continuity of health and social care on release.
- Women benefit from evidence-based health services which are safe and accessible and which maintain decency, privacy and dignity and promote their wellbeing.
- Patients are treated with respect in a professional and caring manner which is sensitive to their diverse needs, by appropriately trained staff.
- Women are aware of the prison health services available and how to access them.
- All women receive information about health promotion and the control of communicable diseases [20].

Analysis of the data was undertaken on NVivo 12 using thematic analysis [33]. The data were inductively coded initially by one member of the research team, then checked by another member of the team, resulting in 24 open codes. These were discussed and reviewed by all members of the team resulting in a number of more substantial, cohesive themes.

### Findings

This section reports the thematic categories derived from data analysis. Where appropriate quotations have been used to illustrate key points and issues. The prisons have not been identified in the reporting of the findings.

### Health promotion models and approaches

Health promoting activities were taking place in the majority of prisons. This ranged from the simple provision of information in the form of leaflets and displays of literature to a more structured calendar of activities across the year in some prisons which could also mirror the national campaigns taking place in the community:

*“There was also a schedule for promoting national health campaigns.”* (Prison 12)

These included presentations, awareness raising events and an educational sexual health programme focusing on lifestyle choices. A small number of inspection reports described the use of peer support with ‘health champions’ providing ongoing support and information for the women.

In a small number of institutions, Inspectors described the *‘healthy lifestyle’* support that was available with a focus on exercise and healthy eating. The majority of inspection reports made reference to smoking cessation services in response to the implementation of the smoking ban in prison in 2017:

*“The prison had been smoke free since September 2017, aided by well-planned smoking cessation support, which was still available.”* (Prison 3)

All of the thirteen inspection reports referred to the health screening programmes employed within the institutions. These included screening for blood-borne viruses, cancer and hepatitis as well as immunisation programmes and systems to manage communicable diseases:

*“Effective systems were in place for the management of communicable diseases. Women had good access to age-appropriate screening programmes, including for breast cancer and blood-borne viruses, as well as to immunisations.”* (Prison 10)

Reproductive health was also a key focus of the services provided to women with sexual health support available in the majority of institutions, including the provision of barrier protection. The reports revealed that barrier protection was not universally available in a number of prisons, frequently not advertised, and available only from health care staff:

*“Sexual health screening and treatment were offered and barrier protection was available, but not well advertised.”* (Prison 12)

In some prisons, barrier protection was only available to women going out on temporary licence or being released. A small number of inspection reports described the information provided to women around menopause, and one inspection report described the *‘excellent’* care provided through the perinatal and maternity pathway used in the prison including antenatal education and postnatal support services.

### Whole-prison approach to health promotion

A joined up approach to health promotion was only cited in a minority of these reports where health promotion was *‘an integral part of the prison’s well-being strategy’* and a comprehensive plan informed by wider national and local initiatives was employed. However, the level of partnership working between the prison and the local primary care and mental health care providers was widely noted in the inspection reports. This constructive working relationship was facilitated through regular strategic meetings which provided effective oversight and governance of the health care service:

*“Commissioners and health providers attended quarterly contract meetings where they could identify and act on key issues.”* (Prison 5)

In a small number of inspection reports an explicit reference to a lack of a whole prison approach to health promotion was made by Inspectors:

*“There was no strategic prison-wide plan for health promotion or any named lead staff member responsible for this area of work.”* (Prison 2)

This resulted in health promotion activities that were *‘too limited’* or provided on an individual basis.

### Democratic inclusion

Around half of the prison inspection reports indicated that the women had an active role in the management of their health while in prison. Some institutions had formal processes in place which allowed the women to take part in consultations through patient participation groups and forums. In some prisons there was a prisoner healthcare representative on the health council and therefore part of the consultation process. There were concerns noted by inspectors of one prison that the consultations were not regular nor did they involve prisoners from across the whole prison. In one prison a new patient participation group was set up as a result of the inspection there.

Surveys were another method used to gather the views of the prisoners and in some cases these had led to impacts on the delivery of services:

*“Effective patient engagement, with regular health improvement groups and surveys, has influenced service delivery improvements.”* (Prison 8)

But it was also noted that the issues raised through these channels could be *‘raised repeatedly without being properly resolved’* and an example of surveys being completed in one institution but then not evaluated was described.

A confidential complaints process was in place in nearly all of the institutions, offering a route through which women could raise individual issues and concerns. In the majority of prisons the complaints process was managed appropriately with a polite and timely response:

*“Health care complaints were managed through a confidential system and passed on to individual health care managers...Investigations were sensitive, addressed concerns raised and provided clear responses.”* (Prison 11)

However, in several prisons the system was not confidential, responses were slow, and women were not fully aware that the system was available to them:

*“Patients submitted complaints through the central hub, which was also not confidential. The complaint responses we sampled had been investigated properly and were polite, but information on how women could escalate their complaint was not always clear.”* (Prison 10)

In a small number of institutions the inspection resulted in a new confidential system being put in place.

### Inequity

In the majority of the prisons inspected the relationship between prisoners and staff was described as *‘caring and compassionate’* with a sense that the staff *‘knew their patients well’*. There were examples of the needs of women being met through appropriate onward referral and an understanding of the impact of wider inequalities on some women with appropriate use of capacity assessments to ensure women understood their treatment, and interpretation services used where women did not speak English.

However, the inspection reports from a small number of institutions illustrated that the needs of some women remained unmet. Specific examples provided in the reports were the needs of older women or those with long term conditions for whom there was a lack of care planning and involvement of the women in the management of their condition:

*“...we found not all patients with complex needs had a care plan. This meant not all staff were aware of individual prisoners’ care needs.”* (Prison 2)

In some prisons the lack of health-related information available in a range of community languages was noted by inspectors. In one prison, information in other languages was available only on request which also excluded those for whom English was not their first language:

*“Health service information was available in reception but it was only in English.”* (Prison 1)

In one prison health information was not available in an easy read format, and in another the information about health services was only given to women who had not been in the institution recently.

### Environment and culture

In the majority of inspection reports it was noted that the welcoming and clean environment of the health care centre was a strength of the service with comparisons made to the delivery of similar services in the wider community:

*“The health centre operated similarly to a community practice, with a reception desk and an open waiting room, and was a welcoming area.”* (Prison 4)

The majority of institutions were described as having a *‘cohesive staff group’* where, if vacancies were unfilled, agency staff were used on a consistent basis to offer continuity in provision from a team with an appropriate mix of skills. A good range of services were accessible seven days a week in most institutions, though one report noted that the service was *‘not suitable for those requiring 24 hour nursing care’* and one institution was only processing applications for health care services on week days. This, however, was reviewed as a result of the inspection.

For a small number of institutions, the Inspectors also noted other adverse environmental factors. For example, delivery of clinical services taking place in multiple areas leading to clinical and logistical challenges, or clinical areas not being as clean and tidy as other areas in the prison:

*“The health centre was located over two floors and comprised clinical rooms, inpatient beds and several offices. On the ground floor, inpatient beds were on the same corridor as the busy outpatient area, which was inappropriate.”* (Prison 7)

Some level of integration of the health care services with the rest of the prison was described in a small number of inspection reports with regular contact between prison managers and service commissioners.

## Discussion

This paper sought to explore how health promotion was undertaken in the female prison estate, with findings derived from the analysis of inspections of women’s prisons in England and Wales. While this methodological approach does not claim to provide a comprehensive overview of current activity, it does draw upon a well-respected evidence source – prison inspection reports – which use a range of methodologies to inform their conclusions.

The notion of equity is high in relation to addressing the needs of people in prison, but even more so in respect to women in prison [16]. One of the criticisms of the health-promoting prisons movement has been its failure to offer a more nuanced and sophisticated approach [22]. No two prison settings are alike and health practitioners and academics should be conscious of the diversity that lies behind the apparent homogeneity [34]. Even within the female prison estate the diversity of women is extensive – the analysis here showed that some of the women’s needs were not being met. This was particularly the case in those not speaking English as a first language and older women. This has been noted elsewhere in regard to the ‘double-disadvantage’ that some women in the criminal justice face [15].

The approach to the promotion of health was aligned to a medical approach and with a preoccupation with ‘lifestyle’ – including smoking and sexual health. In regard to the former approach, screening and vaccination of people in prison has often been a central pillar when describing health promotion activity. Whether this is for health benefits, per se, or for security and safety more broadly is challenging to disentangle. Nonetheless, previous studies have identified just how efficient and effective health services can be in relation to disease prevention in prison [6]. The focus on lifestyle was prominent in the reports with a focus on sexual and reproductive health – there was some excellent practice reported, but this was seen to be inconsistent and largely dependent on the prison. The availability of barrier protection was an example of inconsistent practices across the female prison estate. Such criticisms that health promotion is not consistent across the prison estate is well-established [35] and seen here in regard to women’s health issues.

The notion of a whole-prison approach is consistently cited as being critical to establishing health promotion within any health-promoting prison setting. The recognition that the impacts on health in prison are manifold and therefore need to be addressed by a range of stakeholders is clear – this means that health promotion is *more* than healthcare and should consider all aspects of the environment and culture of the setting. Examples shown of unclean facilities and clinics which were not ‘fit-for-purpose’ was suggested in the data. According to the data from this study, the health-promoting prison was not strategically considered or with clear plans for delivery. Leadership was seemingly lacking in this regard and therefore it was clear that such a commitment to ‘whole-prison’ action was neglected. This is somewhat surprising given the benefits that can derive from whole-systems working [36] and the commitment from government that prisons should be adopting more strategic approaches to health promotion in prison [27].

The data demonstrated examples whereby women in prison had democratic leverage within the structures and systems in the prisons. This, in some cases, shaped service provision positively and could be conceived as an example of community empowerment [37] where women in prison had a legitimate say over how services and structurers could be shaped. Commentators have argued that values such as empowerment are central in creating health-promoting environments – with ‘control’ over determinants of health being crucial to enable people in prison to rehabilitate and address their health needs [30] . Empowering people in prison is often misunderstood and deemed in some cases to be ‘dangerous’ [38]. However, the data here suggested that services improved as a consequence in engaging women in prison in democratic structures. Such features of female prisons should be commended and align cohesively with the theory and philosophy of the health promoting prison. Unfortunately, such instances were not universal and descriptions of ‘tokenism’ were noted in democratic processes. More concerningly, where women in prison wanted to complain about their service or treatment these were not dealt with confidentially or in an expedient way. As noted previously, addressing these matters are essential:

*“It is important, however, that there is a legitimate outlet for grievances for prisoners. The organisational culture in prisons makes it difficult to complain, and the closed nature of the institution adds to the potential for abuse of power.”* ([39] , p.340)

Such instances whereby the voice of people in prison could not be heard seemed to dilute the positive democratic and inclusive aspects that were present in some of the prisons.

To advance both the concept and practice of the health promoting prison, a more detailed analysis of prison settings is recommended and more sensitive policy and practice is encouraged which reflects the remit and function of the prison [40]. To date, much analysis has clumsily homogenised people in prison or indeed the prisons themselves. This seems a major oversight given the crucial importance of context and that ‘one size fits all’ approaches rarely lead to desirable outcomes. The health promoting prison movement has, implicitly at least, focused on the needs of men in prison and this has been to the exclusion of the female prison population. This does lead to several challenges and the potential for exacerbating health challenges faced by an already marginalised and vulnerable group. In the UK, there are signs of a much stronger policy approach to gender-sensitivity in the criminal justice system [15] and while this does not focus on the health-promoting prison per se, it suggests a willingness to recognise differences that underpin those who come into contact with prisons. Internationally, the health of women in prison is a very low-priority [41] with some suggesting whether health-promoting prisons for women globally is an impossibility [4].

## Conclusion

This study has made a number of claims, but also has limitations in its design. First, the analysis is based on inspectors’ views of the prison, which can be overly negative [42]. Second, the study focused on one discreet section of the prison reports and it may be that other areas of the report may have revealed more activities and programmes commensurate with health promotion in prison [7]. However, that does seem unlikely given the recent criticisms that a ‘whole prison approach’ to health promotion is far from a reality [27]. Features of the health promoting prison could have been seen elsewhere in other areas of the inspection, but not reported in the sections analysed. Further research would be required to determine if that was the case, using the perspectives of prisoners as a primary methodological approach [43]. Finally, inspection reports are, by design, cross-sectional and only provides a snapshot in time of activities. Notwithstanding these limitations, the paper suggests that there is some way to go before conditions in women’s prisons could be described as ‘health-promoting’ although there are some examples of individual prisons demonstrating good practice. Given the progressive nature of UK policy in regard to the health-promoting prison [7, 9], but the seeming inactivity of action in practice this raises some concerns in relation to policy implementation. In addition, internationally, where some countries have no clear strategy or ‘will’ for health promotion in prison [4], the situation is likely to be significantly worse with the prison systems generally failing women’s health needs.

## Data Availability

Reports are freely available here: https://www.prisonsinspectoratescotland.gov.uk/publications
